# Supplementary material for: Capturing Russian drinking patterns with the Alcohol Use Disorders Identification Test: An exploratory interview study in primary healthcare and narcology centers in Moscow
Source: PLoS One. 2022 Nov 10;17(11):e0274166. doi: 10.1371/journal.pone.0274166 (PMC9648709; doi:10.1371/journal.pone.0274166)
Supplement: S1 Appendix — (DOCX) [file pone.0274166.s001.docx]

## S1 Appendix. Standardized interview script for the qualitative expert interviews (to be delivered by the interviewer).

*[To be delivered verbally by the interviewer]*

Good day,

My name is ___________. I work in the European Office of the World Health Organization in Moscow. Together with Russian experts, the World Health Organization is conducting a study in which we want to develop a questionnaire that could quickly inform doctors and medical professionals whether their patients might consume alcohol in a risky or hazardous way. This is done to help health care professionals to improve their services and to provide better care. In order to achieve this, we conduct anonymous expert interviews with medical professionals and patients [primary care / specialized medical care] in which we want to find out how certain questions about alcohol consumption are perceived by doctors and patients and what they think about them. If you don't mind giving me 15-20 minutes of your time, then I would like to ask you some questions.

**Informed consent**

*[To be delivered verbally by the interviewer]*

The interview will be conducted anonymously and for research purposes only and will last 15–20 minutes. You have the right not to answer any question(s) and to interrupt or terminate the interview at any time. Do you agree with this procedure?

*[The interviewer waits for oral informed consent]*

**Interview guide for the qualitative expert interviews**

1. Do you think that questions about frequency of drinking (current AUDIT question #1) could tell us whether someone has problems with alcohol or not? [Is frequency of drinking an indicator for alcohol problems?]

- Yes
- No

*Follow up question:*

Why do you think so? Please elaborate.

Do you think that the maximum amount of alcohol a person drinks in one setting (defined as 24 hours) in the last three months could tell us whether someone has a problem with alcohol or not?

*Follow up questions:*

Why do you think so?

Do you think it is important to ask this question to assess alcohol problems, if only three questions about alcohol consumption can be asked (i.e., is it one of the three most important questions in order to determine problems with alcohol)?

1. a. **[For patients only]**: How much alcohol did you drink when you consumed the most during one occasion [*[If needed, interviewer clarifies that by “occasion” we could take* *24 hours to have a clearly defined period*]? [Please indicate in bottles of beer, wine or vodka?

How often did you consume this amount of alcohol in the course of the last 30 days?

How old where you when you started to consume alcohol [age of drinking initiation]? And how old are you now?

*Interviewer note:* check how the respondents answer to an open format question and in case they do not use terms like “standard drink” or other standardized quantifications, please check if they believe that the usual AUDIT is feasible! Make sure that the interviewee understands that the last question refers to age of drinking initiation and not the first time when the person have ever tasted alcohol.

2 b. **[For HCP only]:** How long have you been working as a health care professional?

How long have you been working [with patients] on the topic of alcohol consumption in your practice as a health care provider? [i.e. What would you say, how much experience do you have in asking your patients about their alcohol consumption?]

Did I understand correctly that you are already working with the AUDIT (The Alcohol Use Disorders Identification Test) or have been working with it in the past?

*Follow-up questions:*

If so: for how long?

What are your experiences with the test so far?

Do you use this test regularly?

Would you consider it to be a good test? Why? *[If needed, interviewer clarifies that by “good” we mean useful for everyday work with patients]*

When you look at your daily work, what do you think are certain barriers or problems with this test in your daily practice?

3. There are studies on problematic drinking in Russia that suggest that the following indicators could tell us whether somebody has a problem with alcohol or not:

Having one or more episodes of “zapoi” in the past year (a period of 2 or more days of continuous drunkenness when the person is withdrawn from normal social life)

Twice a week or more: occurrence of excessive drunkenness, hangover, or going to sleep at night clothed because of being drunk.

Consumption of surrogate alcohol (alcoholic products officially not intended for drinking, such as colognes, lotions, alcohol for technical/industrial purposes)

What do you think about these questions? Are they helpful in telling us whether somebody has a problem with alcohol or not? Why do you think so?

What other questions about alcohol use could be used to inform whether a person has alcohol problems or not? What are your ideas?

What do you think marks/characterizes harmful or hazardous drinking? What drinking behaviors lead to negative health outcomes and social consequences?
