# Supplementary material for: Capturing Russian drinking patterns with the Alcohol Use Disorders Identification Test: An exploratory interview study in primary healthcare and narcology centers in Moscow
Source: PLoS One. 2022 Nov 10;17(11):e0274166. doi: 10.1371/journal.pone.0274166 (PMC9648709; doi:10.1371/journal.pone.0274166)
Supplement: S2 Appendix — (DOCX) [file pone.0274166.s002.docx]

## S2 Appendix. Original Russian translation of the Alcohol Use Disorders Identification Test, using standard dinks (back-translated into English).

Source: [[52](file:///C:\Users\neufeldm\Downloads\Interview%20guide,%20materials,%20transcripts.docx#_ENREF_52)]

**AUDIT**

**Please fill in the box for your answer**

| 1.How often do you drink alcoholic beverages?  (0) never  (1) once a month  (2) once a week  (3) 2-4 times a week  (4) 4 or more times a week | 6. How often during the year did you have to get drunk in the morning to feel good after drinking the night before?  (0) never  (1) less than once a month  (2) once a month  (3) once a week  (4) daily or more often |
| --- | --- |
| 2.How many doses do you usually drink when you drink?  (0) 1-2  (1) 3-4  (2) 5-6  (3) 7-9  (4) 10 or more | 7.How often during the year did you feel guilty or regretful after drinking?  (0) never  (1) less than once a month  (2) once a month  (3) once a week  (4) daily or more often |
| 3.How often do you use 6 or more standard doses of alcohol in the single occasion (booze)?  (0) never  (1) less than once a month  (2) once a month  (3) once a week  (4) daily or more often | 8.How often during the year have you been unable to remember what happened the previous evening because you were drunk?  (0) never  (1) less than once a month  (2) once a month  (3) once a week  (4) daily or more often |
| 4.How often during the year have you felt that you could not stop drinking?  (0) never  (1) less than once a month  (2) once a month  (3) once a week  (4) daily or more often | 9.Have you or anyone else been injured after drinking alcohol?  (0) no  (2) yes, not this year  (4) yes, this year |
| 5.How often during the year have you been unable to do what depends on you because of alcohol consumption?  (0) never  (1) less than once a month  (2) once a month  (3) once a week  (4) daily or more often | 10.Was a friend, doctor, or other health care worker worried about your drinking or suggested that you limit your alcohol intake?  (0) no  (2) yes, not this year  (4) yes, this year |
