# Supplementary material for: Capturing Russian drinking patterns with the Alcohol Use Disorders Identification Test: An exploratory interview study in primary healthcare and narcology centers in Moscow
Source: PLoS One. 2022 Nov 10;17(11):e0274166. doi: 10.1371/journal.pone.0274166 (PMC9648709; doi:10.1371/journal.pone.0274166)
Supplement: S3 Appendix — (DOCX) [file pone.0274166.s003.docx]

## S3 Appendix. Alternate updated version of the Russian translation of the Alcohol Use Disorders Identification Test, using a frequency-volume table (back-translated into English).

Source: [[53](file:///C:\Users\neufeldm\Downloads\Interview%20guide,%20materials,%20transcripts.docx#_ENREF_53)]

**AUDIT - the Alcohol Use Disorders Identification Test**

Circle the number that is closest to the patient's answer.

**1. How often do you drink alcoholic beverages?**

(0) never

(1) once a month or less often

(2) 2-4 times a month

(3) 2-3 times a week

(4) 4 or more times a week

**2. What is your typical alcoholic beverage intake on the day of drinking?**

|  | Standard portion | Vodka (ml) 40 vol% | Fortified wine (ml) 17-20 vol% | Dry wine 11-13 vol% | Beer (bottle) 5 vol% |
| --- | --- | --- | --- | --- | --- |
| (0) | 1 or 2 | 30-60 | 75-150 | 75-150 | 250ml-1 bottle |
| (1) | 3 or 4 | 90-120 | 225-300 | 300-400 | 1.5-2 bottles |
| (2) | 5 or 6 | 150-180 | 375-450 | 500-600 | 2.5-3 bottles |
| (3) | 7 or 8 | 210-240 | 525-600 | 700-800 | 3.5-4 bottles |
| (4) | 10 or more | 300 and more | 750 and more | 1000 and more | 5 and more bottles |

**3. How often do you drink more than 180ml of vodka (450ml of wine) in one drink?**

(0) never

(1) less than 1 time per month

(2) 1 time per month (monthly)

(3) 1 time per week (weekly)

(4) daily or almost daily

**4. Over the past year, how often have you found that you are unable to stop drinking?**

(0) never

(1) less than 1 time per month

(2) 1 time per month (monthly)

(3) 1 time per week (weekly)

(4) daily or almost daily

**5. How often in the past year have you not done what is usually expected of you because of drinking?**

(0) never

(1) less than 1 time per month

(2) 1 time per month (monthly)

(3) once a week (weekly)

(4) daily or almost daily

**6. During the past year, how often did you need a drink in the morning to recover from a previous heavy drink?**

(0) never

(1) less than 1 time per month

(2) 1 time per month (monthly)

(3) 1 time per week (weekly)

(4) daily or almost daily

**7. Over the past year, how often have you felt guilty and/or remorseful after drinking?**

(0) never

(1) less than 1 time per month

(2) 1 time per month (monthly)

(3) 1 time per week (weekly)

(4) daily or almost daily

**8. How often during the last year have you been unable to remember what happened the day before because of your drinking?**

(0) never

(1) less than 1 time per month

(2) 1 time per month (monthly)

(3) 1 time per week (weekly)

(4) daily or almost daily

**9. Has your drinking ever caused injury to you or others?**

(0) never

(2) yes, but it was more than a year ago

(4) yes, during this year

**10. Has it ever happened that your relative, friend, doctor, or other healthcare professional showed concern about your drinking or suggested that you stop drinking?**

(0) never

(2) yes, but it was more than a year ago

(4) yes, during this year
