# Supplementary material for: Capturing Russian drinking patterns with the Alcohol Use Disorders Identification Test: An exploratory interview study in primary healthcare and narcology centers in Moscow
Source: PLoS One. 2022 Nov 10;17(11):e0274166. doi: 10.1371/journal.pone.0274166 (PMC9648709; doi:10.1371/journal.pone.0274166)
Supplement: S4 Appendix — (DOCX) [file pone.0274166.s004.docx]

## S4 Appendix. Translated transcripts of the qualitative interviews used for the original analysis.

### PHC Patients

**PHC_Patient_1 [Male, 73 years old, age at drinking onset: 17, did not consume alcohol for the last 19 years ]**

*“I consumed on Saturdays. On Sunday I did not drink because I had to go to work. [...] I was drinking a lot. [So you used massively on Saturdays?] Yes, massively. [Massively - how much was that?] Well, a bottle. [A bottle of what?] Vodka. “*

*“[Answering question 2 of new version of AUditscreen.org with SDs and frequency table] Vodka, and then also beer. In general, I drank fortified wines too. [And all that at once on Saturdays?] Yes. [...] [So you drank vodka, fortified wine, and so all at one time?] No, not at one time. [Wel, I mean during a Saturday?] Yes, during a Saturday. [That is, during one Saturday, you drank* ***and*** *vodka* ***and*** *fortified wine* ***and*** *beer]. Yes, this and beer. [...] [So you understand this instrument? Or something needs to be changed?] No, nothing needs to be changed. [So you understand everything?] Yes, yes ... ”*

**PHC_Patient_4 [Male, 30 years old, age at drinking onset: 13, consumes alcohol from time to time]** *“Well I could drink a bottle of strong alcohol [...] [And strong alcohol is what exactly?] Vodka, whiskey, rum. Whatever it is. [Is this 0.5 then?] 0.7 I think I drank. But I felt bad afterwards .”*

*“[Answering question 2 of new version of AUditscreen.org with SDs and frequency table] That is, what is my usual dose of drinks when I drink at the weekend? Well, probably one standard drink. [That is something from this?] Probably even the second option rather. [So in principle, you find your way around in this questionnaire? So if I ask about the standard portion, is it clear here?] Yes, yes. [So with the explanation her, that the standard portion/standard drink is so and so much vodka and so on] ... Yes. I have the second option, 3 or 4 standard drinks then."*

**PHC_Patient_6 [Male, 61 years old, age at drinking onset: 30 ,did not consume alcohol for the last 15 years]** *"Earlier? Well 150, 200. [Sprits?] Spirits. [On one occasion?] In one day, not at once. On one occasion is a lot. [Well, that is, the daily dose is roughly speaking] Well, yes, then 150 ”*

**PHC_Patient_7 [Female, 51 years old, age at drinking onset: 17, drinks sometimes but much less than earlier]** *“I do not consume anymore, at my age. And in my youth ... Well, I wouldn’t say that an entire bottle, but it’s on holidays only, like if we are going somewhere. Generally I didn’t drink, but if I drank it was not an entire bottle, maybe 200 grams .I mean: all in all, while you are sitting, while you are having fun, maybe 200-250 grams. It's all in all: you drink, you eat, you talk, you dance, right? This is not about getting drunk. Well, so that, there were no situation that I was lying somewhere drunk or so - this was not the case. [What exactly do you mean with 200-250?] Well, vodka, maybe wine. [...] Well, a glass. Well, it's ... how many hours are there? For 5, for 6, that's as long as you sit. For example, if there is a wedding or a birthday party. Sometimes we are meeting with friends. Well there we might have a glass. Just for fun. To relax. [And when vodka is drunk, then wine is drunk as well?] No, no mixing, personally from my own experience: do not mix. This is already bad. Either wine or vodka. Only one thing. [...] Now I’m already of a certain age and have certain health problems. But I drink, of course. Who is not drinking these days? And before it was the same. For fun a little bit, to lighten the mood. Only on holidays. With occasion. I was never drinking for no reason. ”*

**PHC_Patient_8 [Female, 66 years old, age at drinking onset: 17, drinks sometimes but much less than earlier]**

*“You know, I all my life I have been working with men. Well, there they always have some kind of holidays, something they celebrate at the factory. But categorically, I cannot stand vodka at all. If I drink it a little bit, then I feel bad. And they usually either bought weak wine for me or ... And now I remember that I drank, maybe 200 grams during this whole celebration. I felt so bad, I barely reached [from one metro stop to another]. Then I went to the toilet and spent the whole evening there. It was very bad. [It was the maximum amount that you ever drank?] Well, yes, there were maybe 200 grams for the entire day that I could have drunk. And maybe less. Because I can't stand vodka at all, I can't. It is like medicine. [And other alcoholic beverages?] And others…A little sparkling wine, but I usually go to sleep after this. And as I’ve said: I’m not a big fan. Immediately, I fall asleep and that’s it. [...]”*

*“[Answering question 2 of new version of AUditscreen.org with SDs and frequency table] I would state that I consume fortified wine because I do not like dry wine. Either sparkling wine or fortified. Something sweet ... [And how much do you drink of it?] Well, 75 grams. [So in principle, if the questions are asked in this form, that's when it is explained how much of what ...] Yes, it is fine. [So, in principle, you can find yourself in here?] Yes, yes, this is ok.”*

**PHC_Patient_9 [Male, 55 years old, age at drinking onset: 16, did not consume alcohol for the last 15 years because of self-reported alcohol dependence]**

*“[And how much did you usually drink?] It was always different. It depended. I could drink a bottle. [A bottle? That is 0.5?] Yes, yes. And I could also 0.7. But there were also times, were, of course, I could also drink nothing. [And the maximum amount you drank on a day?] Well, let’s say 0.7. [Was it during one day?] Yes. [And did you go to work during time?] Yes, I could go and also drink at work. And I worked and there was nothing wrong about this. Well, in Soviet times, this was more or less in the order of things. You just knew that you had to work. ”*

*“I think this indicates the absence of money. So for instance, if you have 1,000 rubles in your pocket, you can go to the store and buy normal vodka. But you can also buy windshield wiper fluid for 15 rubles if you only have 30 rubles. So if you have the shakes and you need something to ease the hangover (“opokmlelyat’sya”), you won’t think long. You just go and buy it. […] The question that you ask is whether surrogates are consumed because of alcoholism or not. Buy if you have money, you will buy normal things, so I think that the question is not really correct. Surrogates are consumed simply because of the lack of money. Yes, of course, this question is about alcoholics, about alcoholism. Yes, it’s about that but I think it’s not really just that because an alcoholic, if he has the money, will go and buy other things. If someone is an alcoholic it’s about easing the hangover and if you have the money you buy normal vodka and if you don’t, you buy windshield wiper because you don’t care. [So if I understood you correctly, this is not about being an alcoholic but about being a poor alcoholic?] Yes! Yes, exactly!”*

**PHC_Patient_10 [Female, 56 years old, age at drinking onset: 15, drinks sometimes but much less than earlier]**

*“Well, firstly it was a long time ago, when I was maybe 20 years old, during my days of being student, mauby I was 23 years old there. Well, I drank maybe half a bottle of wine. This was probably my limit. And then, it was not that I was dead drunk. But simply: ah, we have fun, come on, we are students, we are having a good time! I’m not particularly that ... And if I drink, then I need my wines to be sweet, more like this. I probably have tried vodka once, maybe twice in my life. Since it is bitter and, as they say, not tasty, it was of no interest to me.”*

*“[Answering question 2 of new version of AUditscreen.org with SDs and frequency table] I understand and see all these questions. But I don’t know... For me, this is generally not of any relevance. And even for those for whom this is relevant, this is all garbage they will say. I do not know ... ‘How often do you drink more than 100 milliliters in one drink ...’ I do not know. It seems to me that those, who [do not drink excessively] may tell you something, but those, who already do - it is generally not important for them and they will not count all this. Somehow I am skeptical about this. [...] Me, I don’t drink beer at all, dry [wine] quite rarely, fortified - well, what I mean is probably fortified is what I drink. These sweet wines, yes. Such as Massandra, Cahors, the ones that are expensive and of high quality. Probably this then. [But the fortified wines, that's just the point, there is more alcohol in them] Well, I drink just one glass, maybe two in total. [So you mean particularly sweet stuff? Because fortified wine has a volume of 17-20%] Well, then I don’t know what is meant. [Therefore, I ask you exactly what you mean] I am not an expert on this issue, I cannot answer this way. [...] Well, this is just my case. And my case is specific. I think that most cases can be found in there and suit this scheme. ”*

**PHC_Patient_11 [Female, 56 years old, age at drinking onset: 15, drinks sometimes but much less than earlier]**

*“Well, I don't drink alcohol at all. Only if there is some kind of holiday, like a wedding or something like that - half a shot glass then. I first drank a shot. First one, second one and then I was not feeling good at all good. A cousin of mine - I look at her and she is drinking little by little, little by little. And I ask: ‘And what are you doing?’ And she says, ‘If I get drunk at the very beginning, then I will not see anything from the feast.’ And I also started to maneuver. To drink little by little, the whole wedding was like this then. [That is, a shot of vodka?] No, I have a little there ... a third of the shot glass there. So little by little. In general, it turned out maybe 100 grams. No, not 100 grams. Well maybe 150 grams was maybe what I drank. [...] This is at a big event, at a wedding, or maybe at an anniversary, something like that. [...] A shot of vodka, yes. And so I - for a holiday you can have a little wine, for instance [...] How much is a shot glass? Well, probably 100 grams. No, shot glasses are small glasses. And in total, it turns out to ne probably somewhere well ... a shot glass of 100 grams, well, a half, if everything together for the whole evening, for the whole holiday. Well, the wedding happens once, twice a year. But I general, I do not drink. "*

**PHC_Patient_12 [Male, 81 years old, age at drinking onset: 10, still consumes frequently]**

*“We do not drink alcohol, here in the family. So - on a holiday, a little something there and here. Generally, when I have employees at work, they have hard physical work, it’s in the garden, you see, it’s in a standing position, there’s some kind of lactic acid that is produced, it’s necessary to neutralize it somehow. Well, they pour themselves a glass, 30-50 grams. Well and they also pour me [a glass]. But I drink it without much pleasure. [...] This is once a day, even not every day, but probably so, 50 grams, not more. Well, of course, this is either cognac or… Well, in general, I’m gagging, but slowly I drink it. [That is, they drink a little bit during their work in order to make it physically easier for themselves?] Yes, yes. Well, it seems that some scientists have said [to the employees] that you have such processes [going on in your body] and that you need to drink alcohol in order to neutralize them. [...] [To the question about the maximum quantity per day]. Well, I never drink this way. If I buy a bottle, I put it in the bar, it stands there and I forget about it.”*

*“Although for myself, I would have enjoyed using something like ‘Madera’ or ‘Sherry’, bit I don’t have the money for that. Good stuff. I know perfectly well that it would be good for a person to consume alcohol at the level of 12 grams per day of pure alcohol, well, it depends, in general, from 12 to 20 grams, but unfortunately I ... These people who use within these limits live longer. This was on the Internet, there were articles, there were studies, that is, there were a lot of people there, 20 thousand or so, and there they asked how much you use, how much you drink. And they found out that someone drank a lot, someone drank a little … and lived [those who drank a little, lived longer than those, who did not drink at all]. But again, those who consumed a lot, they mostly died not so much from alcohol, but from other troubles they died. Like got hit by a car, or something else. ”*

**PHC_Patient_13 [Female, 31 years old, age at drinking onset:20, does not drink at the moment]**

*“For a day ... I don’t know. Earlier, when I was young, I could have beer at some concert probably yes, a lot. There, maybe 8 bottles per day, this is what I could drink. [And how often did this in the last three months] Never, I have one pregnancy after another, no.”*

*“[Answering question 2 of new version of AUditscreen.org with SDs and frequency table] Well, this scheme is so relative. A person can drink one thing, another thing, a third thing - a person will not keep track of it all. Firstly, he does not remember how much he specifically drank, and secondly, he can mix or not mix, and this .... I think it is not necessary. [That is, there is the problem of mixing alcohol?] Of course, this is the first issue. Secondly, if a person is intoxicated, he will not remember for sure. This is useless, I think, the question. A person cannot answer objectively. Maybe this is a good question if he would directly answer correctly and accurately, but the person simply cannot answer for sure. [...] If a person drank beer, then went on and someone else has invited him to have something else, then he got the idea into his head that he wanted to go on and drink something else. Nowadays, this is how it happens in Russia. "*

**PHC_Patient_15 [Male, 72 years old, age at drinking onset: 17, does not drink for the last 3 years]**

*“This should be translated into normal language. I myself understand how many 10 grams of ethanol are. I remember how much alcohol is there and how much vodka is, I myself understand this. But it is necessary to translate this for everyone because one cannon figure it out immediately. Well, it is better to say something like .... A wine glass. 50 grams of vodka. Then everything will be clear. And if there will be 10 grams of ethanol instead, then one needs to think. [So if explain to you right away that we are talking about a glass of vodka, a glass of wine and so forther...] Yes, it will be faster to answer like this!”*

**PHC_Patient_16 [Female, 71 years old, age at drinking onset: 20, does not drink for the last 10 years]**

*“[Can frequency tell us whether a person has problem with alcohol or not?] Probably. [...] Frequency leads to habit. A habit leads to alcoholism. [So we should ask about this?] Of course! But how to ask? ”*

*“[Can the maximum amount consumed tell us whether a person has problem with alcohol or not?] “Well maybe. If a person drinks three bottles.... Yes, I think so. [That is, the maximum volume is worth asking?] Of course. ‘How much can you drink? ’One will say a bottle. And the other will say a glass. [...] For drinking- one needs a lot of time. You drink today, tomorrow you have to recover from drinking, the day after tomorrow you are more or less okay. All this takes time. I feel sorry for all the time spent on this and we didn’t have the time - children had to be raised when we were youn, and then it was difficult to go to work [after drinking].”*

*“No, I do not drink, of course. I feel bad after it. I do not like this. When I was still working at another job, there were still some gatherings, and so ... [When was it?] Well, 10 years ago. [Well, so this means that were drinking about 10 years ago and now you don’t drink at all?] Yes, yes. [But you said you drank in your youth. So what maximum amount could you drink there?] [Listen, these were student years, what could we have drunk there? I do not know, some t red bormotukha [cheap fortified wine] probably. But not vodka. But very little amounts. One still needed to get home. So no, no.”*

*“[Answering question 2 of new version of AUditscreen.org with SDs and frequency table] Listen, I remember now: I drank beer. In the late 90s. When I was going from work, I was buying a bottle of beer. [So you drank a maximum of one bottle of beer?] Of course just one.”*

**PHC_Patient_17 [Female, 72 years old, age at drinking onset: 20, drinks from time to time]**

*“[Can frequency tell us whether a person has problem with alcohol or not?] Frequency – this means how often someone consumes? Then of course! Well, of course [...] Both, frequency* ***and*** *quantity. You can drink half a glass of dry red wine at dinner — and that's okay. Or you can drink a bottle..”*

*“[Can the maximum amount consumed tell us whether a person has problem with alcohol or not?] “No. I do not think this is an indicator. There are different stressful situations. I judge it by myself - sometimes I can drink a glass and sometimes I can drink three sips, depending on my condition. [...] Although there are many opponents of this judgment, but alcohol reduces stress. If a person is in some kind of stress, they need some more. And if you are here to relax, to enjoy the fact that you drink good wine – then you need less. But this does not mean that it is alcoholism. Then then it will be necessary to formulate the following question: ‘In connection to what do you drink?’ [You mean in terms of situation?] Yes, to find out the situation! It can be - Well, there are certain situations, for instance if guests came from somewhere. Or some anniversaries happening in a row, then it’s like: you have to, even if you don't want to [drink]. And then there are situations that a person [drnks] just here, alone. So it is all about the circumstances and the reasons. ”*

*“I could drink a bottle of dry wine. It was probably six months ago, on the countryside. Well, but this was in the process, over the course of several hours. Not that I sat down and, sorry for the expression, knocked myself out. No, it was in the process of a conversation, communicating with others. That is half a liter of wine. Red dry wine. ”*

### PHC Doctors

**PHC_doctor_1**

*“[1. Do you think that questions about frequency of drinking could tell us whether someone has problems with alcohol or not? / Is frequency of drinking an indicator for alcohol problems].*

*Well, of course [...] The frequency of [alcohol consumption] probably correlates with the risk there, right? Therefore yes”.*

*“[2. Do you think that the maximum amount of alcohol a person drinks in one setting (defined as 24 hours) in the last three months could tell us whether someone has a problem with alcohol or not?] Well, if a person can count for three months, then probably it can tell us this. [So it depends on the person's ability to count?] Well, if you can count this, you can probably also correlate. [...]*

*Probably the more often you drink and also more in terms of volume, then there is certain risk, yes? This is logical. Right? Well, in this regard, and yes. [...] If a person remembers exactly how much he drank in three months, then probably… If it is possible, why not? If he himself counted and said: ‘I drank three liters of wine in three months’ -So what? Yes maybe. [...] And in one day – whether he can estimate this or not, I do not know. If you ask for three months, then he should definitely say: February 3, how much he drank, right? [...] Well, I am not an expert in narcology, I cannot tell you. I do not know if a person drinks this or that much in three months, as far as it is a risky consumption for him. I cannot say, I do not know. [Just to clarify once again: when patients talk about their consumption, they count in liters?] Why? It depends on how we ask. The way we ask – this is also the way they answer. [And how do you ask?] Well, we have an AUDIT questionnaire; we ask how much they uses - frequency of use and quantity. How many alcoholic units/servings, like one or two. We tell them what an alcohol unit is and they tell us how much they consume. [And do people understand this concept of alcoholic units?] Well, we tell them what it is. That it is 150 milliliters of wine or 40 grams of strong alcohol there.*

*“[What difficulties or obstacles did you encounter in working with AUDIT?] None at all. We ask questions - they answer. There are four questions only. And that's all.”*

**PHC_doctor_2**

*“[1. Do you think that questions about frequency of drinking could tell us whether someone has problems with alcohol or not? / Is frequency of drinking an indicator for alcohol problems].* *I think yes. [...] I think that these should be questions about the frequency and amount of alcohol consumed, respectively, at a time, since this will allow us to estimate the real, so to say .... well, not real, but indicative use. [...] Frequency is one of the important criteria here. Of course. Frequency, well, maybe multiplicity, I would say. Yes, multiplicity. [...] Well, it is what we have in the AUDUT right now, there we ask how many times a day, a week, so many times a month. That is: how often.”*

*“2. [You mentioned some daily dose or for some period, that is, apparently, intensity too?] Well, yes, yes, for the day, yes, intensity, too, the amount consumed at a time, for one alcoholic excess, let's say yes, or for one day. Maybe indeed a daily dose, since alcoholic excesses can be quite lengthy or maybe vice versa it can be interrupted during the day, for example, if we talk about people who chronically consume alcohol, so here ‘in one sitting/on one occason’ [as the answer category] probably will not work. And we need to ask precisely enough, so yes, probably in a day, this seems to me to be the most accurate. So yes, this would reflect[their consumption].”*

*“[Usually people understand [questions], yes]. [...] Well, as far as daily consumption is concerned, there are no questions in generally [...] Questions arise usually in relation to what to do, how to answer, if they drink alcohol… well in different quantities, how to average that amount to name some specific dose. Well, many say, for example, that at one day they can drink five glasses of wine, and on another time they will drink one glass of wine - and what should they answer in this case, how to calculate the average amount. So, this is probably a really interesting question that has no definite answer, at least for me, yet. [...] Usually we ask what is more typical for [patients], that is, what situation is more frequent. That is, something will be considered more typical and something less typical behavior for a person. And so they will have to answer and choose a more typical option.”*

*"I usually ask them to express [their consumption] in a certain amount of milliliters of a drink, and of course I calculate the doses myself because it’s difficult for patients, as a rule. Because there are many options, it’s clear that it is not difficult to calculate as such, but if you account for all alcoholic beverages existing, it will be a very big question and its essence will be lost. Here we assume [the following categories]: fortified wine, ordinary wine, vodka, etc. There ... there are other alcoholic beverages, maybe less often consumed, but nevertheless also consumed by some patients. Therefore, I ask them to estimate/report this in milliliters of a particular drink, which is typical for them, and I recalculate myself to the standard drink, then in the second step. [...] It’s more convenient for me, I think that it’s also more accurate than the option where a person has to estimate the amount, meaning that he has to calculate the number of drinks himself. Sometimes it is easier for a person to say that he drinks, I don’t know, half a bottle of wine than to decide how many glasses. Because the glasses are different as well, and again: you can pour 100 milliliters into a glass, you can also pour 150 in the same glass, if we are talking about wine. Therefore, here people are better oriented and seem to be more confident if we formulate it in this way. [In milliliters?] Yes, yes. Milliliters of a certain beverage. Not in portions/standard drinks, but in milliliters. [I will just clarify: is it then that you ask absolutely open asking a question?] Yes, yes. That is, for instance ‘What is the average amount of alcohol consumed in this particular typical case. And I propose to give me just some figure in milliliters of some alcoholic beverage, one that will be convenient for you to use as an example. Which is more typical for you in terms of consumption’. [And in your experience: is this the most convenient option for you and the patient for mutual understanding?] Well, yes, at least it raises fewer questions than if you offer [the patient], for example, a flash-card with options, how many standard drinks they use, and what's the standard drink is, so and so many milliliters of this, so and so many milliliters of that. It takes time and not everyone understands it completely. [So did you observe difficulties in patients?] Yes. [So it is easier for them to do this in milliliters?] It seemed to me, yes. This is easier and, in general, faster, which is also important in terms of screening, because time is very limited in finding out what is going on. [And how much time do you spend on this, on this AUDIT-C?] AUDIT-C, yes. Well, about a minute and a half. "*

*2.“[Do you consider the question on the maximum alcohol volume consumed so important to include it in the questionnaire?] No, I probably don’t think so. [Can you explain a little? Before that, you said that the frequency was ...] Yes, the frequency [is imporant], but it’s about how exactly to ask in terms of what the maximum intake was, if... if you already know that all the alcohol he had drunk was so much more than necessary. Then what difference does it make what the maximum intake was? I think what is important is whether this is a single dose of alcohol, normative, or whether this is systematically more. But the upper limit, no, I do not think that this should be clarified. [...] If I understand the question correctly, then we look at the period of three months. A man drinks there, for example, a bottle of vodka on one sitting/occasion. There, each occasion is a bottle. And on some occasion he drinks a bottle and a glass. But what is the point for us to know that he drank a bottle and a glass this one time, if we already see that he use excessively. In this regard, I do not see why we need to ask for the maximum dose. [...] Rather, it makes sense to identify the norm for a person. [That is, not the maximum dose, but the usual one?] Yes, yes, you can express it like that. [That is, if I understand you correctly, it is important for you to identify the frequency of consumption and the typical dose consumed?] No, no, typical only if it is about excessive use. It is still necessary to reveal the gradation, how much is used. [But not the maximum volume in this case, because you do not think that it is so important] Probably not.*

**PHC_doctor_3**

*“There [2 question] it was presented with an example of some alcoholic beverages ‘How often do you drink for example 30 milliliters of spirits’. And in brackets there is brandy, or for example, vodka. Or there is this count: ‘How many alcoholic beverages do you drink?’ And then this explanation goes in brackets in the form of 30 milliliters of sprits, 100-150 milliliters of dry wine and 300 milliliters of beer. This is counted as one portion/standard drink in this questionnaire. Accordingly, it’s clear for the person, he is oriented. First, he can estimate the alcoholic beverages he chooses [consumes typically] and in what amount he drinks it more often. [...] This is listed directly in the questions, in brackets - what amount of alcohol you consume for example at a time. An in brackets - there goes this decoding/explanation of what is meant by this word “portion/serving”, that this is 30 milliliters of spirits, for example.”*

*“Since the question is asked in portions/standard drink and the answer is written down by me specifically, so it is I, who writes the number down and enter it into the questionnaire, this allows for that the person can speak to me in different ways. For example the person can say ‘Yes, I usually drink for example 2 glasses of beer. A glass of beer I usually have is 500 milliliters.’ Accordingly, 500 [and two glasses] - it is clear to me that this is 1000 then. Well, I quickly recalculate this in my head- how many servings, based on the fact that a standard drink is 300 milliliters, respectively, these are 3 and something. Well, there are answer options - it is necessary to tick one. Not to enter a specific figure, but there is one servings, two servings, three servings or four. In this case we talk either about 1- 2 standard drinks or 3- 4 standard drinks, there are specific boxes as answer options for this. And I tick the option of 3-4 standard drinks, respectively. Well, or either the person himself counts it in his head and says: well, yes, I’m probably drinking a glass of brandy for the evening, for example. And so he says: I have 30 milliliters of brandy there. Accordingly, I drink one portion and I tick the box with one standard drink. [According to your experience and your impressions: can you calculate this right away, on the spot? Or is it difficult?] No, no, this is generally quite simple, not difficult. Most often, everything is clear and very specific. Well, firstly, because there are already those predefined answer boxes, I don’t need to write down any specific figure, I just choose from the existing boxes, there are 1-2, 3-4 and I ticks this at the level of these boxes. I do not have to think, is, it’s very clear. And if a person says that they drink a liter of beer it’s not that I have to calculate it, like how many it is specifically in terms of standard drinks specifically and recalculate that 300 milliliters there are three portions and something something - I don’t need to calculate a specific number, I just need to select a box. Well, this is probably logical that is not worth this specific calculation at all, that there is plus or minus a tenth of a share / portion, probably this is not so important. [So if there is already some kind of a ready template, then it is convenient for the medical worker?] Yes, that is enough. And it does not take much time at all. Well, in principle, people understand. We never had any issues, in principle, connected to this Well, that is, if a person remembers [...] well, there are issues with [remembering] frequency sometimes. But, actually the questions on alcohol were quite specific in there [the research project].”*

*“[So how did you ask the second question then?]Well, just ‘How often ..’ ‘How much do you drink in one sitting?’ And our people, some of them asked: ‘And what is this -one sitting/one occasion? I started at 3 o'clock and it was a feast and then we went somewhere else, sat down and then we went on, we did this and that and then we walked there and went somewhere else to see the fireworks, went back and forth and so on..’. It’s like if we have a wedding for example or some kind of events that take place for 4-5 hours. Is this considered one sitting? There were some questions on this. Well, this is quite rare, because all such events, they are not regular. We tried to ask everyone in the same manner, in our questionnaires we are talking about some kind of regularity, that's exactly what you have, what you can say for every day, for every week, and so forth. [...] Well, I still tried to explain what ... Well, it is clear that we do not take all these weddings, feasts and all that into account, but rely on an average, we equate it, I do not ask about yesterday's specific day, I don’t ask about a particular day, ‘How much did you drink on this particular day, when you had this feast/celebration?’ We ask: in general, on average, as usual.”*

*“Our question was asking for ‘one occasion’, so I regarded it as in one sitting. There was no specific question for the day. I tried to explain to the person how I understand it myself. But there were actually very few [who needed clarifications]. Many understood what it was. The question was: - ‘How much alcohol you drink at a time?’. For myself, I understood this as part of one meal, for example. That is, at one time, - for instance someone sat down for a meal, began to drink and now he got up when he was done and left. Be this a dinner occasion, be it something else- whatever you want. Here it is. And I had someone who said that for dinner, for example, I drink one glass of wine. So I wrote it down as one standard drink, well, there is one glass of wine for example. [...] When it was not clear, I concluded that it would be in one sitting. That is, you sat down, started eating, drinking, some kind of feast/celebration. Let it be even a holiday, if a person is sitting with a people in a holiday, too, he, too, can probably control this process, yes, you can sit there ... No, in fact there were no issues with that, this is quite understandable. People responded quickly enough, there was no particular hitch.”*

**PHC_doctor_4**

*“It seems to me that the main difficulty is to make your respondent understand that he is actually the one, who needs this/profits from it, so that he will be sincere and give truthful information.I had practically no difficulties in presenting this information [to the patient] or interpreting it. Well, my situation may differ from others because I know the science behind it. I know what it is needed for. I know that this is a good tool and that it helps to identify problems that are not possible to identify with any other tool. That the test is needed specifically for general practitioners and not narcologists. [So you think AUDIT is a good tool?] Yes. [...] We have something to compare it to. We have a very large amount of information on completing this questionnaire in the framework of clinical examination. That is, at first there was the CAGE questionnaire. We received on the average one and a half percent, meaning that this was the prevalence of a positive [CAGE] score, so one and a half percent for Russia. In different regions in different ways, there were 0.5 for example for some. Now, we literally just recently received the database of people who filled out the questionnaires in the framework of dispanserization, which included the AUDIT as well, a short version of it. Thirty thousand people filled this out. And we got 6.5% positives for women and 8.1% for men. Significantly higher. Therefore, I believe that it works even in this version, when people fill in themselves. Not even when experts are interviewing them, but when they fill in test by themselves. And I think that these numbers are actually low, they should be higher.”*

*“No, one should not just ask about a SD [without explaining]. In any case, it is necessary to tell people what it is. Or immediately [...] immediately translate the SD into the usual volume. [...] Se we immediately present a completed version so to speak. That is: the most common drinks in Russia are wine, beer, and strong alcohol. And we immediately present the amount of alcohol that is contained in these drinks. Well, more precisely the opposite, that is, the amount of alcoholic beverages that correspond to the SDs, which are contained in this questionnaire. Either the questions is immediately unfolded [...] so the test is preceded by this information that one SD contains 30 milliliters of strong alcohol, 250-300 milliliters of beer, and 100 milliliters of wine — this is one SD. How many SD do you drink? [...] It is convenient ... This option is best understood by people. It seems to me that it is best to give information ... Even in those guidelines on AUDIT, which the WHO offers, there are two options for completing the questionnaire. I mean the long version, although - what's the difference anyway? The option of self-completion and the option when the interviewer does it. And here there can be different approaches. When a person does the AUDIT alone, then everything should be clear immediately, everything should be understandable at the first glance, so that the person understands. When the interviewer fills in, there is always the possibility of explaining something. So with this option [the interviewer], you can specify the SD. This is what I know for sure, this I understand for sure. And yet we, when we even conduct trainings, we say that self-completion is not the best option. It is still better to complete this with the interview method.”*

*“Still, the concept of a SD is needed. It must be kept in the question and explained to the patient. Firstly for what it is. So this concept has also an educational function. Many people believe that low alcohol drinks, for example, are better than spirits. They believe that they contain less alcohol, so to speak. That is, they do not understand that the main component of an alcoholic beverage is ethyl alcohol and that it is important for us to understand the total amount of alcohol consumed. And they should understand this. And therefore the concept of a SD must necessarily be in the question for patients who [fill out the test by themselves] ... Well, in an interview too. To not just tell them that these are alcoholic beverages, that how much beer... or whether you drink such or such amount of different alcoholic beverages. Then again, different people, especially men, drink different alcoholic beverages during the week. That is, they can drink beer and then spirits, so it is more convenient to convert this into some standard units. For this, in principle, the concept of a standard unit was introduced in the first place.”*

*“If we are talking about the third question [...] when we are talking about the frequency of not [sic!] heavy drinking. That is, we must understand how often a person consumes more than six or five SDs, by the way, in different versions there are different numbers of these standard drinks. In any case, it is necessary - a person must understand ... Asking the third question, we cannot but mention this term. That is, to ask the third question simply in quantities of alcohol, we cannot do that, it turns out too stupid. Have you ever used more than ... Or how often do you consume more than 180 milliliters of vodka or 600 milliliters of wine during one event? In any case, it’s easy to do when we’re figuring out normal consumption, it’s understandable there, a person can say, and there’s no need…. The specialist can translate everything into SDs. He asks - how much is usual. Well, usually I drink half a bottle of vodka. Or: I usually drink two bottles of beer. I usually drink half a bottle of wine. Everything is OK. They [the patient] said this, I translated this into SDs, filled out the questionnaire and it’s okay. But as for the third question where we have to find out this maximum amount, then it seems to me inconvenient to ask a question [with quantities] ... There you just won’t ask. What is the maximum amount of alcohol drunk occasionally?”*

*“It is very difficult. [...] Yes, in Russia there is no such thing as the concept of “binge drinking”, here it is necessary to use English. No concept of “occasion”. That is, it is also not clear - what it is, how do we call it? We are trying to solve this situation in a way that we are calling it a type of “one event” or “some kind of event associated with the use of alcohol”. That is, one cannot immediately talk about one day for sure. We are talking about an event of some kind and not just for one day. What is the maximum amount of alcohol you drink in one day? No, not during one day, but during one event. ”*

### Narcology patients

**Narcology_Patient_1 [ Female, 34 years old, age at drinking onset: 17, currently in treatment]**

*“The thing is if you ask alcoholics, you don’t even need to show them this. Every alcoholic will tell you it’s more than 10. But if you are asking not alcoholics but people at their early stages, so of course they might stop and reflect...But the problem is that not every person will tell you the truth. […] I think this depends on anonymity and how honest the person is. ”*

*“In 24 hours? So about a liter of vodka. ”*

*“I think people will look more at these [volume] numbers here. Because the 10 grams- no one will calculate this. I think if you show bottles here or glasses, it will make it much easier to answer this question. Because I would not understand what this means: three to four and five to six standard drinks. Because it’s not clear what you mean: either litres or grams or…[So in either case we need some form of table or showcard or…?] Yes, some way of correcting this. Because people won’t understand this. Or you won’t understand our people.”*

*“So I have come to an understanding for myself that when I start to drink this means that I start to drink. Because these are different things for an alcoholic and for a person who is not an alcoholic. We [alcoholics] would understand this in our own way, while people who are not alcoholics will understand this in completely different way. They would tell you that they drink once a month, like for instance a couple of glasses during a birthday celebration. But for us, this is more about drinking. Like really drinking, when you start to drink and have a zapoi and so forth and this is different. […] So again, this and the other question [on number of standard drinks] are not correct for alcoholics. Now, all the people who are in here for treatment, they will just tell you that they drank the maximum amount and that’s it.”*

*“Yes, even when a person drinks the first day and on the second day has to drink because of withdrawal, because you need to drink to ease the hangover, that’s it…This will continue as a zapoi for sure when a person does not know how to stop.”*

*“This is really clear. A person who is not an alcoholic won’t drinks colognes. We don’t even to discuss this one here. [So this is quite a clear criterion for alcohol dependence?] Of course!”*

**Narcology_Patient_2 [Female, 33 years old, age at drinking onset: 16, currently in treatment]**

*“So if you present this as standard drinks – it’s not comprehensive for me. But if you present it like this, this is very comprehensive. [So in principle with this show card here you can manage]. Yes, of course, if you have this, then this is clear. [So you could manage this on your own if you had the show card?] Yes! ”*

*“So I could drink about five to six litres [of beer] during 24 hours. […] So I drank about two cans of beer per day, every day.[…] [So right now we ask how much people were drinking per day. Would it somehow influence your answer if we were asking how much you drank per occasion?] It would! I think it’s better how much you drink per day. [Per day or per 24 hours? What do you think?] Per 24 hours. Some people work shifts. Like two shifts rotational patten. Some people have regular 5 working day weeks, some people work shifts. So I think it’s better to ask for 24 hours. Because it can be that someone is working the shift and doesn’t drink but then has two days off and is drinking the first 24 hours and then is trying to make themselves presentable the next 24 hours before getting back to work. ”*

*“So last year I had a zapoi. More than two days, more than a week actually. I was drinking non-stop, you could say around the clock. So I bought a bottle of wine and thought that it’s enough for me. But it wasn’t enough, so I went on and bought another two bottles. I thought that I can just keep it around. But I couldn’t. So this is how this becomes a 24 hour thing. You wake up and you don’t feel well. You drink and you sleep, drink and sleep, drink and sleep. And this went on for nine days. […] So for me this is what a zapoi. You wake up, you drink, you sleep and so on. For me this was nine days in a state of stupor. So basically I don’t remember these days at all. I just woke up, drank and went to sleep again. Just somehow automatically managed to feed my cat. I think it was some subconscious thing that you woke up, had to feed him and then- morning, evening and that’s it. […]. That’s about dependence because a normal person won’t drink this way. […] So when we talk about alcohol dependent people, we’re talking about at least two days [of drinking], even more. ”*

*“[Twice a week or more: occurrence of excessive drunkenness, hangover, or going to sleep at night clothed because of being drunk]. It’s the risk zone. This is not yet addiction but this is absolutely a risk zone. Because I had this when I was not yet an addict. It was some kind of warning sign that I had to stop. [And what exactly? The excessive drunkenness the hangover or…] The excessive drunkenness! But this again depends on the beverage. So for instance when I was drinking beer, I was absolutely capable to undress myself before going to sleep. But if this was strong alcohol, vodka, then yes…it happened that I once woke up still dressed because I passed out. ”*

*“I never tried [surrogates] but you should ask this question [… ] So this is definitely about addiction when people come down to drinking surrogates. […] This is really the rock bottom, not even our unit here, but possibly some other clinic […] for really lower social status people. I think people end up with surrogate alcohol when they no longer can afford alcohol for…When they don’t have enough money for a good quality beverage, they drink the surrogates. So this means that the person is sick. It’s not even dependence, it’s sickness. ”*

**Narcology_Patient_3 [Female, 32 years old, age at drinking onset: 14, currently in treatment]**

*“Well, I did not count of course, but as I’ve said at the beginning I could drink about 2 litres […] of vodka. I prefer vodka.”*

*“Well, the maximum I could drink at once.... I think you can drink two liters. [of vodka]. It depends on the company, on the people with whom you sit and drink. If you are sitting in a normal company, for example with relatives and friends, well, normal friends, that’s one thing. And you drink alcohol for fun. But there are companies where the consumed amount is the same as with relatives, with normal people, but it already affects you in a bad way. And it can have certain consequences, it can increase your drinking. [...] Because there are different companies and they drink differently. The one drink just for fun. And the others drink just drink. Do you get what I’m saying? They are just getting drunk with vodka. That’s the difference. [...]. At first, you start drinking to relax: in nightclubs, restaurants… Then all this gets boring and the gatherings begin at home. You don't have to go anywhere. You just sit and drink. Sit and drink. Sit and drink. And that's all. You even lose interest in other things. If earlier it was fun to drink, then later you just need to drink.. "*

*“The longest zapoi I have ever had was a month. But it doesn’t mean that I drank two litres per day. It was only at the beginning, the first day that I could drink two litres. Then it becomes less and less […] Still, it is a zapoi. Every day you get up and you need to drink to ease the hangover. So during the day you hangover-drink(“opokmlelyat’sya”), but the dose gets lower every day. From two liters to less. But still, this is a zapoi. […] So a zapoi is” to drink and to sleep. To drink and to sleep. This is the main thing. Sometimes some action is going on, for instance the crowd moves from one apartment to another […] you also exchange one crowd for another until you are completely out of it and when it’s about night time you sleep. Then you get up, look for money. If you have money, you don’t have to look for anything, just buy [alcohol] again, sit down and drink until you are piss drunk and then you sleep again. So this is what it is: a bad zapoi. The last phase of alcoholism. […] So I started to have zapoi when I was about 25 years old. This is when I got involved with a bad crowd. And this is when this all started. At about 25. And when these zapois have started. And between them, the sober periods were about one month, I could also hold on for two months, three months, half a year. I’ve never managed to stay sober for a year, half a year was the longest for me before I’ve relapsed again. So you get out the zapi, you hold one and preserve, but you have and feel some kind of premonitions. At first you don’t understand but with every time afterwards after a series of zapois in the course of many years you already understand that before [the zapoi] you get more aggressive, more irritable. Like everything tells you that you really want to drink and then, at the end it’s just one drop, like for instance you feel depressed and down, like something is missing. Seems like everything is fine , but you feel empty and sad. And this is where you relapse. And where the zapoi happens”*

*“This is the last stage of alcoholism […] So I’ve said: as long as you have money, you drink the normal stuff. Even if you drink a lot, but you still manage to drink the normal things. But if you have no money. Like at the beginning you have money, then you spent everything on alcohol, then you need something to do about this because you’re in the zapoi. Then you start to look for things. Do you remember, a while ago they were selling these small bottles with hawthorn? I’m not talking about the medicinal tinctures from the pharmacy but these bath lotions. Everyone was drinking the. Probably you’ve heard when […] people died in Irkutsk. [Where these sold in kiosks?] They were sold everywhere. In every shop. I’ve been also drinking it. It was sold in all stores, even in grocery stores selling foodstuff. For instance, I live in a small and there, we knew that we could get this in different spots where this was sold. And this costed only 25 rubles. So we were just looking for money, collecting it, went to buy it, diluted it and drank it and our [withdrawal] condition improved and we felt better. When we got worse, we were looking for money again to buy these small bottles. […] So it was exactly with these small bottles that I had my biggest zapoi of one month. Then, when I was already feeling better, I just learned by chance that in Irkutsk 17 people died. Some [counterfeited] batch came out on purpose. But I think they did this on purpose because of the competition. Because it’s not only these small bottles. The thing is that alcohol is not sold after 11 PM anymore. And in our town they also sell this in the houses, in every second house they are selling either ethanol or samogon or they are giving you this on credit. Some people accumulate debts of five thousand, three thousand, four thousand roubles… Different people, different debts. So they give this to them, on credit and not…And it’s cheaper than in the stores and you can buy this at night. […] Now they sell this more from the houses. I no longer see these small bottles. […]. They sell in houses – samogon or ethanol. [So just to summarize: the problem in itself did not disappear? People find alternatives?] It did not disappear and yes, they find ways, they do.[So it’s still there?] Yes it’s still there and still works the same way. At the houses and apartments. People sell from their private homes. [So this means it went rater to the apartments? From the kiosks and stores it went to homes?]. Yes, yes…Samogon.[…] Samogon distillery equipment. […] Samogon to be sold. […] Samogon for own use. For instance, when I was a child, my grandparents were making their own smaogon because they needed to mow the fields, so they were giving these to the guys helping and also for own consumption, but I’m talking specifically about sale here. There are really long lines for these, people come and buy. [So it was like this before the poisoning of 15-16 and…] This is how it was and how this remains. [So they banned the hawthorn but other things…] Other things remained. There is no more hawthorn but all these other things, they stayed. [And you were saying that after 11 PM you can’t buy alcohol anywhere. So did this somehow had an influence on how…] No. I would say no. I think it hasn’t. There is always a way to find it. Even more so in a small town, where everyone knows each other, where you know people, for instance you know that one saleswoman in this shop and she can sell it to you and just check this at the cash register in the morning. People find their way regardless. […] Even looking at myself, I can tell you from own experience, that If you really want it, you’ll always find a way. [So even now, in the year 2019, regardless of all the alcohol control policies introduced, all the bans and…] It’s still all the same. At least in the villages and small towns. I can’t say how things are in the cities. Maybe things are the same in small cities. […] Colognes. People drink colognes. […]. In my case this was because I ran out of money, I had nothing left and in order to get rid of the shakes, I needed something. And the cheapest thing for 25 rubles was [hawthorn lotion], this was the cheapest to find. Again, we are talking about a small town here, so you just go around scrounge money. ‘Give me two roubles. Give me five roubles.’ Once you managed to scrounge up enough money you go and buy it. This is because it’s so cheap.”*

**Narcology_Patient_4 [Female, 38 years old, age at drinking onset: 17, currently in treatment]**

*“I don’t understand this. Not clear. [So from your point of view, should we be asking in bottles then or what?] Most likely so, yes.”*

**Narcology_Patient_6 [Male, 54 years old, age at drinking onset: 21, currently in treatment]**

*“Please write this in grams. For instance, 50 grams, 60 grams 100 grams and so forth. Do not write this in milliliters. You might measure this in milliliters measurement, but people will not understand this. […] [And why grams specifically?] Well, because in Russia we are used to drinking in grams. 100 grams, 200 grams, 300 grams, 400 grams, half a liter.”*

**Narcology_Patient_7 [Male, 45 years old, age at drinking onset: 14, currently in treatment]**

*“[What is the largest amount of that you have drunk in a day?] Oh, that’s a good question, I don’t know. There must be six bottles of vodka. Per day. [0.5 each?]. Yes, 0.5. [So about three liters]. Yes, three liters. [...] As part of a zapoi I had. “*

*”This is already the bottom, the absolute rock bottom. […] You really hit the rock bottom when you do this. […] Some of our pharmacies, they sell pharmaceutical alcohol illegaly. I think it has about 95%. So in reality this alcohol is meant to be used as a hand disinfectant for the surgeon before the surgery. So at some point, this thought shot through my mind ‘What the hell am I doing here? What am I drinking?’ […] So I somehow realized […] that I’m already at the brink of an abyss here. […] This was at a pharmacy about two years ago.”*

**Narcology_Patient_8 [Male, 31 years old, age at drinking onset: 20, currently in treatment]**

*“This is a bit unclear. [So you need some explanation here?] Yes.”*

“*About three liters of vodka [So this was in the course of 24 hours?] Yes […] I had a zapoi. I had some problems so I just decided to step out and to drink and so it happened that I just spiraled into drinking, which is why this needs treatment now.”*

“*This is already death. [Why?] Because everyone knows that if you drink technical alcohol you will get blind. [Okay, but we are talking about ethanol-based alcoholic products here, not methanol]. Not methanol? But still, it’s counterfeit. I’ve tried it once and I felt very very bad afterwards. Worse than if you drink normal vodka. […] It was a counterfeit, like a bottle of Henessy whiskey that I’ve bought for 150 rubles. […] Some people drink hawthorn and [other medicinal tinctures], even some of my friends do and they are happy with this because they don’t have the money for vodka. They get into withdrawal and they are happy that they have this. [So would you say that these people have an alcohol problem?] I would say that they are goners. “*

**Narcology_Patient_9 [Male, 63, age at drinking onset: 16, currently in treatment]**

*“This is difficult to convert. I even saw such a questionnaire in a polyclinic once and there, you simply don’t have the time for this. You stand at the reception desk and fill out all these forms, a lot of forms, and there, you don’t have the time to think about all these standard drinks and glasses. [So basically you already filled out such a questionnaire]. Not this one here but something similar, as part of a medical checkup during dispanserization. […] There were two pages about alcohol at the very end. […] So still, I think you should not ask this question as based on ethanol units. It should be in glasses. Still, everyone understand this – small and big glasses.”*

*“So before I was admitted [to the clinic] my intake was somewhere between 1.5 liters to 300 ml per occasion of strong spirits, now it was about 150 ml. […] Samogon or whiskey or something like that. As for vodka, there are a lot of counterfeits out there. ”*

*“[Twice a week or more: occurrence of excessive drunkenness, hangover, or going to sleep at night clothed because of being drunk]. This person most likely drank ethanol/non-beverage alcohol. I can even tell this from my own experience, when I was drinking non-beverage alcohol. So I never fell asleep behind the table. But it happened that I passed out because of as for non-beverage alcohol, like sitting behind a table with food and everything and then I wake up just like this, fully dressed and everything. Doctors told me that one should never drink non-beverage alcohol, especially men, so I really believe them. ”*

*“A lot of people dilute colognes with water and drink them. Especially those, who come out of prison. They are really good at this thing. […] So this is really a pointer and highlights [problems with alcohol]. So I drank non-beverage alcohol […] tis was because it was there and no other alcohol was available. So I decided to try this. [So because of availability?] Availability, yes. I was told that this is medicinal alcohol and I got a canister for something, I don’t remember what, but I needed this for some reason and it was there […] It was not of very good quality although they told me that they got it from a hospital. But I finished it very quickly, like for some technical things needed in the house, but also in particularly because of that [drinking], I diluted it, tried to get more water into this. […] Of course, only a person with alcohol problems will drink non-beverage alcohol.”*

### Narcology doctors

**Narcologist, familiar with the instrument and screened/interviewed a big amount of people with the AUDIT in the primary health care setting (using the AUDIT-C version) Voice_30**

***Narcology_doctor_2***

*“The AUDIT is a good instrument, also in working with patients [it] can be used for psychotherapeutic purposes. You perform an AUDIT and then interpret its domains and tell the patient whether he has a dangerous consumption pattern or dependence and you intervene. [...] The AUDIT is needed, we have it included in clinical recommendations, in the protocol associated with hamrful consumption [...] In ICD-10 there is such a diagnostic category such as harmful use, so in particular when we have cases with as harmful use or when we have a dubious borderline case, the AUDIT is quite necessary. So when we are talking about harmful use, it is important for us to quantitatively measure this degree of adverse effects, and in this case the AUDIT zones work quite well. And by defining these zones, we can perform more targeted brief interventions. [...] There are outpatient departments and there is this category of harmful consumption. Moreover, our narcologists are represented in such medical organizations as health centers, prophylactic centers they are entrusted with this task and they need the AUDIT, there, they work with practically healthy populations, relatively healthy.”*

*“The AUDIT has problematic areas, which we will talk about later, but it fully reflects the Russian realities. It should be understood that one of the main advantages of AUDIT is its cross-cultural character and the possibility of its application on different populations with obtaining similar results.”*

*“Particularly for screening purposes, screening of so called healthy individuals, [the first three questions] are extremely important. Particularly these first three questions.”*

*“There are difficulties [with AUDIT], though not so much for us as much as for the patients. [...] Neither in a professional environment nor among consumers of alcoholic beverages- we simply do not have the concept of a standard drink, it is very underdeveloped. Standrad drink is a very complex concept. Often, when you ask a question, the frequency is clear, but the number – there the main questions arise. We had special flashcards displayed with certain portions of alcohol, colored and laminated which show: “This is one drink. This is beer, alcohol. How much do you drink? Let's count!” Standard drink- people do not understand. And in general, the problem of the standard drink, it is big one. For instance: WHO Drink and Scandinavian Drink, they are different. […] So if, for example, in the UK they indicate the number of drinks on bottle labels in terms of safer consumption and any Brit understands what a drink is, in Europe they understand what a drink is, our Norwegian colleagues have not encountered any problems with this, then we spent time explaining and establishing what a drink is. If you administer [the AUDIT] just as a self-questionnaire, then 98% of the population will not answer correctly the questions related to drinks. Or it should be explained in the questionnaire, which is quite difficult. At the same time, to explain this is not about giving the formula ‘10 milliliters of pure alcohol is…’ plus this 0.7 conversion factor ... Well, they [the screened patients] will not count. Especially if you work with older people - they will just dismiss the study. Therefore, it is necessary to give specific doses: ‘How often you drink so and so many doses of beer, so and so many doses of vodka …’.But then the survey becomes visually very overloaded. But this is the only way out if you want to use it as a self-questionnaire. There is no other option. Because the culture of the drink will not be developed very soon.”*

**Narcologist, familiar with the instrument and screened/interviewed a big amount of people with the AUDIT in the primary health care setting (using the AUDIT-C version) _Voice_31**

***Narcology_doctor_3***

*“AUDIT was created for primary care physicians who do not know our criteria for the dependence of ICD 10, the criteria psychiatrists and narcologists operate with. And people at risk of developing possible complications and risks [in relation to their alcohol consumption], they do not come to psychiatrists-narcologists. We get to see people, who are already sick, who already have and addiction. Or there are people with harmful use. [...] But there are just a few narcological patients admitted with harmful use, it’s, little, this is probably a small percentage.”*

*“[About how the AUDIT can be applied in PHC setting since the interview partner has worked as a GP previously ] This means that we will have to find out about tobacco use and alcohol use in the history of the disease. That is, we have to warn ... Although what are we warning about? What kind of confidentiality will be maintained? I doubt it, because taking into account the fact that we immediately fill in the history of the disease, and then in the history of the disease immediately we have: name, surname, passport. Our patients are a little wary of this. Because they all expect some kind of consequences, sanctions and so on. Especially those who work. Because after some time, it will turn out that they will come back to receive some kind of doctor's opinion/form on their fitness to work for civil service, and there is this information in his file that he is misusing alcohol, and so on. [...] Initially, we need to think about how to make it in such a wat that our recommendations do not just shake the air, but really will become recommendations. Not just ‘Drinking is bad and so on.’ Or, the doctor should explain to the patient that he has atrial fibrillation due to the fact that he is misusing alcohol and if he stops drinking alcohol and starts taking appropriate therapy for the heart rhythm disorder, then maybe everything will be fine. But, strictly strictly, it is not allowed to use psychoactive substances, including alcohol. Then the patient may listen. [So initially it is necessary that everything would be clear from the start ...] What to do with it, yes!*

*“In addition, the question also arises with these standard drinks. [...] We had pictures for a clear understanding of what we actually ask there. That is how much roughly one standard drink of alcohol is. We said 10 grams of pure ethanol, although there is still a conversion factor. To be honest, we didn’t bother about it that much and I don’t think that it is so important. We simply showed that it was there: a glass of wine, a small bottle of beer, 0.33, a shot of vodka. For our patients, these drinks are the most common, they are clear and understandable. But if young people hang out in clubs and drink cocktails there and they don’t know, and also we don’t know how much alcohol there is in one cocktail, how the bartender mixes it - nobody understands, here’s how to count? It will also be difficult. By the eye. [...] If [AUDIT] is administered in the form of an oral survey, that is, the doctor asks these questions, then that image/flashcard will be enough, just like ours. Maybe you will make it more interesting or it will be generally an electronic version [...] there you could make a calculator for these standard drinks. But this is for advanced and young people or if it is an oral survey. But if it is self-completion survey, it will take a lot of time and there will be a lot of mistakes because the patients do not always understand what is wanted from them and some of them do not want anyone to know about their problems. Even a doctor.”*

**Narcologist, familiar with the instrument and screened/interviewed about 15 people with the AUDIT in the primary health care setting (using the AUDIT-C version) Voice_32**

***Narcology_doctor_4***

*“So about the difficulties with the standard drink: people often do not remember. Especially if a person consumes a lot it is very difficult for him to indicate a typical/regular dose and on the day of drinking. That is, if alcohol is consumed frequently, then it simply falls out of memory, especially if we ask you to indicate some time ago as well. The dose is very often difficult to specify, especially if this is not the same beverage. There are people who drink only spirits, then it is easy to specify. But when they drink alcoholic products of ambiguous quality, when it is strong alcohol and beer [mixed together], then it is difficult to point out, especially if we ask you to indicate which dose was drunk. Especially when it happens often, as I say. The more people drink, the more difficult it is to specify all these parameters, from my point of view. They are waiting for further clarifying questions. [...] From my point of view, when there is a table/overview on the type of and quality of drinks of alcoholic beverages, then this is faster understood and processed than words/descriptions. [So there were question about this formulation of ‘dose’?] ‘Dose! What is as dose? Do you understand?’ Of course, it depends on the strength of the drink, on the quality of the drink; this is not a simple question. [...] That's what I want to say: it is also not easy for the interviewer to ask these questions.”*

*“I first started using the instrument, it was in a private [...] clinic where patients were turning to, who had not yet had a pronounced dependency. Since the clinic was multidisciplinary, we treated patients mostly with somatic problems and general practitioners were working there. So general practitioners, when interviewing patients, they were also generally assessing if a person consumed more alcohol than they should. And besides, if it was established that if alcohol use was the cause of or contributed to the somatic illness with which the patient had presented in the first place, the doctors referred then and accordingly told him to go to a narcologist at the same hospital, and so the patient was sent to the ward in which I worked. There I began to apply the AUDIT test.”*

*“When I was preparing, when this test was presented to me, I noticed that the standard drinks are different. In Russia, the concept of a standard drink is generally very relative. Because as I understand, in the West people can count in drinks, because they often drink alcohol in a bar and it is easier to count. In our culture, people more often consume alcohol at home. When they drink at home with friends/in groups, it’s harder for them to count. And it was necessary to adapt the drinks that are indicated in the test, to translate along with the patients, to calculate how much standard drinks they actually consume.”*

***Narcology_doctor_5***

*“The problem with that is if you ask someone in primary healthcare ‘What is your maximum alcohol intake per occasion’ they will tell you something like ‘100 grams’. But the problem is that this way, you won’t get the person’s real maximum intake. Because maybe he had these 100 grams about three times or four times. So, this is not valid then. It’s better to assess this like we.”*

***Narcology_doctor_6***

*“When talking about a zapoi we are always talking about the loss of control. It’s not about situations like for instance New Year, where I am visiting my relatives, we sit behind a table as a big family and of course- we drink there every day. Is this a zapoi? […] For instance, when you have time off and you meet your friend […] and you are going out enjoying your freedom and loosening up and drink every day, but you are not getting drunk. Is this a zapoi? So, for a narcologists a zapoi is always about control loss**.”*
