# Supplementary material for: Capturing Russian drinking patterns with the Alcohol Use Disorders Identification Test: An exploratory interview study in primary healthcare and narcology centers in Moscow
Source: PLoS One. 2022 Nov 10;17(11):e0274166. doi: 10.1371/journal.pone.0274166 (PMC9648709; doi:10.1371/journal.pone.0274166)
Supplement: S1 Table — (DOCX) [file pone.0274166.s005.docx]

## S1 Table. Characteristics of the interviewed participants, according to sub-samples.

| **General and preventive care hospital patients**  Total n =9 (5 males, 4 females)  Age range 19-85, mean age: 62.6, median age: 71  Mean age at drinking onset: 18.1  Drinking status: 8 current drinkers, 1 former drinker |
| --- |
| **Polyclinic patients**  Total n =8 (3 males, 5 females)  Age range 31-81, mean age: 57.1, median age 56  Mean age at drinking onset: 17.5  Drinking status: 5 current drinkers, 3 former drinkers |
| **Narcological patients (different facilities)**  Total n =8 (3 males, 5 females)  Age range 31-63, mean age: 41.7, median age 38  Mean age at drinking onset: 16.4 |
| **Health-care professionals (n=12)**  Preventive or specialized health-care professionals (cardiologists): n= 5  Narcology specialists: n =7 |
