# Supplementary material for: Capturing Russian drinking patterns with the Alcohol Use Disorders Identification Test: An exploratory interview study in primary healthcare and narcology centers in Moscow
Source: PLoS One. 2022 Nov 10;17(11):e0274166. doi: 10.1371/journal.pone.0274166 (PMC9648709; doi:10.1371/journal.pone.0274166)
Supplement: Attachment — Submitted filename: PloseOne answers to the reviewers.docx [file pone.0274166.s006.docx]

**PONE-D-21-18136**

**Capturing Russian drinking patterns with the Alcohol Use Disorders Identification Test: an exploratory interview study in primary healthcare and narcology centers in Moscow**

**PLOS ONE**

Journal Requirements:

When submitting your revision, we need you to address these additional requirements.

1. Please ensure that your manuscript meets PLOS ONE's style requirements, including those for file naming. The PLOS ONE style templates can be found at 
<https://journals.plos.org/plosone/s/file?id=wjVg/PLOSOne_formatting_sample_main_body.pdf> and 
<https://journals.plos.org/plosone/s/file?id=ba62/PLOSOne_formatting_sample_title_authors_affiliations.pdf>

2. When reporting the results of qualitative research, we suggest consulting the COREQ guidelines: <http://intqhc.oxfordjournals.org/content/19/6/349>. In this case, please consider including more information on the number of interviewers, their training and characteristics. Moreover, please provide the interview guide used as a Supplementary File, in English and in the original language.

3. Thank you for stating the following in the Acknowledgments/ Funding Section of your manuscript:

This work was supported through a grant of the Russian Government to the WHO European Office for the Prevention and Control of Noncommunicable Diseases. This funding source had no role in the design of the study, its execution, data collection and analyses, interpretation of the data, writing of the manuscript or decision to submit it.

Please note that funding information should not appear in the Acknowledgments section or other areas of your manuscript. We will only publish funding information present in the Funding Statement section of the online submission form. 
Please remove any funding-related text from the manuscript and let us know how you would like to update your Funding Statement. Currently, your Funding Statement reads as follows:

This work was supported through a grant of the Russian Government to the WHO European Office for the Prevention and Control of Noncommunicable Diseases. This funding source had no role in the design of the study, its execution, data collection and analyses, interpretation of the data, writing of the manuscript or decision to submit it.

Please include your amended statements within your cover letter; we will change the online submission form on your behalf.

4. We note that you have indicated that data from this study are available upon request. PLOS only allows data to be available upon request if there are legal or ethical restrictions on sharing data publicly. For more information on unacceptable data access restrictions, please see <http://journals.plos.org/plosone/s/data-availability#loc-unacceptable-data-access-restrictions>.

In your revised cover letter, please address the following prompts:

a) If there are ethical or legal restrictions on sharing a de-identified data set, please explain them in detail (e.g., data contain potentially sensitive information, data are owned by a third-party organization, etc.) and who has imposed them (e.g., an ethics committee). Please also provide contact information for a data access committee, ethics committee, or other institutional body to which data requests may be sent.

b) If there are no restrictions, please upload the minimal anonymized data set necessary to replicate your study findings as either Supporting Information files or to a stable, public repository and provide us with the relevant URLs, DOIs, or accession numbers. For a list of acceptable repositories, please see <http://journals.plos.org/plosone/s/data-availability#loc-recommended-repositories>.

We will update your Data Availability statement on your behalf to reflect the information you provide.

5. Your ethics statement should only appear in the Methods section of your manuscript. If your ethics statement is written in any section besides the Methods, please move it to the Methods section and delete it from any other section. Please ensure that your ethics statement is included in your manuscript, as the ethics statement entered into the online submission form will not be published alongside your manuscript.

6. Please include captions for your Supporting Information files at the end of your manuscript, and update any in-text citations to match accordingly. Please see our Supporting Information guidelines for more information: <http://journals.plos.org/plosone/s/supporting-information>. 

7. Please review your reference list to ensure that it is complete and correct. If you have cited papers that have been retracted, please include the rationale for doing so in the manuscript text, or remove these references and replace them with relevant current references. Any changes to the reference list should be mentioned in the rebuttal letter that accompanies your revised manuscript. If you need to cite a retracted article, indicate the article’s retracted status in the References list and also include a citation and full reference for the retraction notice.

[Note: HTML markup is below. Please do not edit.]

Reviewers' comments:

Reviewer's Responses to Questions

**Comments to the Author**

1. Is the manuscript technically sound, and do the data support the conclusions?

The manuscript must describe a technically sound piece of scientific research with data that supports the conclusions. Experiments must have been conducted rigorously, with appropriate controls, replication, and sample sizes. The conclusions must be drawn appropriately based on the data presented.

Reviewer #1: Yes

Reviewer #2: Yes

2. Has the statistical analysis been performed appropriately and rigorously?

Reviewer #1: N/A

Reviewer #2: N/A

3. Have the authors made all data underlying the findings in their manuscript fully available?

The [PLOS Data policy](http://www.plosone.org/static/policies.action#sharing) requires authors to make all data underlying the findings described in their manuscript fully available without restriction, with rare exception (please refer to the Data Availability Statement in the manuscript PDF file). The data should be provided as part of the manuscript or its supporting information, or deposited to a public repository. For example, in addition to summary statistics, the data points behind means, medians and variance measures should be available. If there are restrictions on publicly sharing data—e.g. participant privacy or use of data from a third party—those must be specified.

Reviewer #1: No

Reviewer #2: Yes

4. Is the manuscript presented in an intelligible fashion and written in standard English?

PLOS ONE does not copyedit accepted manuscripts, so the language in submitted articles must be clear, correct, and unambiguous. Any typographical or grammatical errors should be corrected at revision, so please note any specific errors here.

Reviewer #1: Yes

Reviewer #2: Yes

5. Review Comments to the Author

Please use the space provided to explain your answers to the questions above. You may also include additional comments for the author, including concerns about dual publication, research ethics, or publication ethics. (Please upload your review as an attachment if it exceeds 20,000 characters)

**Reviewer #1:** This is an important subject area because the alcohol content in a standard drink in different countries varies and it is necessary to adapt that audit questionnaire to local conditions. It is difficult in Russia were the concept of standard drink not exist and even more difficult is the definition of drinking occasion. The first aim of this study was to identify problems with understanding all of the audit items. The second aim was to improve the clarity of those items. A third aim was to explore possible solutions and the existing practices in screening at health care providers. A fourth aim was to integrate experiences of the use of the audit questionnaire. However I have some comments I hope will make the text easier to follow.

1. In table 1 information should be written in the usual way, now it seems not to be a table. Maybe it is necessary with a statistical test between the groups regarding the age.

*A1: We have deliberately provided this information in a table form to give readers at least a rough idea of the different subsamples and the interviewees, who were recruited using a convenience sampling method, as discussed in the limitations section. It does not seem sensible to apply a statistical test to the different groups regarding age given that this is a very small non-representative sample. We also have tried rewriting this section as advised, putting the information contained into text format, but it made the description of the samples even more complicated and less transparent. Therefore, we would like to continue to use the current format (Table 1). If deemed necessary, we can move the table to the Web Appendix, but describing all the information that it contains in written form within the text does not seem to improve readability of the manuscript.*

2. I would be happy to see the citations from the interviews because they are the data which the conclusions are built upon. Now the conclusions standing alone which makes it difficult to control the conclusions for the reader.

*A2: We have now added several citations into the different sections of the manuscript, and we hope that they provide a clearer idea of how we have arrived at the conclusions.*

3. It can be problematic to Translate the concept standard drink into doses without specifying what a dose is and how much alcohol is contains. It is not clear how the volumes are calculated from standard portions in table 4.

*A3: We have clarified in the manuscript (in relation to Table 3 and Table 4) that these items are taken from the two different Russian translations, which are featured on the AUDIT website of John Saunders (*[*https://auditscreen.org/translations/*](https://auditscreen.org/translations/)*). We have identified over 60 different Russian translations of the AUDIT and, in many of them, it was not clear how the standard drinks or the final scores were calculated and there were many other, more serios issues identified (Bunova et al., 2021; Neufeld et al., 2021).*

*The main point of the exercise was to explore how PHC and narcology patients perceive the second AUDIT item when its content is represented in different way. We were not measuring the patient’s actual alcohol consumption or related risks.*

*References:*

Bunova, A., Neufeld, M., Ferreira-Borges, C., Bryun, E., Fadeeva, E., Gil, A., ... & Rehm, J. (2021). The Russian translations of the Alcohol Use Disorders Identification Test (AUDIT): A document analysis and discussion of implementation challenges. *The Intezrnational Journal of Alcohol and Drug Research*, *9*(1), 20-29.

Neufeld, M., Bunova, A., Ferreira-Borges, C., Bryun, E., Fadeeva, E., Gil, A., ... & Rehm, J. (2021). The Alcohol Use Disorders Identification Test (AUDIT) in the Russian language-a systematic review of validation efforts and application challenges. *Substance abuse treatment, prevention, and policy*, *16*(1), 1-14.

Auditscreen.org. AUDIT 2021 Available from: https://auditscreen.org/cmsb/uploads/audit-russian.pdf.Accessed: 26/04/2021 53.

Auditscreen.org. AUDIT (Russian): Приложение C 2021 Available from: https://auditscreen.org/cmsb/uploads/audit-russian-new.pdf Accessed: 26/04/2021

4. Item 3 in the audit Define if the person is a binge drinker or not. But in the Russian version according to table five item three assess maximal amount alcohol a person drinks in a day and how often. It is not comparable with binge drinking frequency. Another problem with measuring alcohol consumption in Russia is there a high number of people who drink unrecorded alcohol that is not assessed with audit.

*A4: As described in this section, patients and healthcare professionals alike reported difficulties in understanding the concept of binge drinking and what was meant by the term “one occasion”. Because of this issue, the interviewer was exploring what “one occasion” meant to different interviewees and, if difficulties in reporting arose, the interviewer clarified that respondents should consider an occasion as being equivalent to a 24-hour period since this was a concept well understood by everyone. This part of the interview was deliberately left open-ended and exploratory in nature to capture the different perceptions and cognitive concepts, as described in detail on pp. 22-24.*

*The same can be said for unrecorded alcohol, which was included in this exploratory qualitative study as an important concept exactly because this is commonly consumed in Russia but not covered by the AUDIT. The results discussed in this article were used in the empirical validation study of the AUDIT in different Russian regions, where specific questions unrecorded was included as well, but the validation results have shown that adding specific items for unrecorded alcohol does not improve the performance of the tool. This is, however, in line with the present results that consumption of unrecorded alcohol is closely linked with alcohol dependence, suggesting that it is worth exploring the inclusion of such items into a Russia-specific addiction scale.*

**Reviewer #2**: The article is very well written and very clear, the study was needed and the adaptations will be useful in research and clinical practice, as planned. A few typos remain, so another look would be helpful (Background section: 'patters' (patterns); ...creation and validation for (instead of of) a Russian version of...

A1. The manuscript was again proofread by a native speaker and we hope that we have addressed all typos and other issues.

We thank the reviewers for their comments and hope that we were able to address all of them. The editing of the initial manuscript has led to an increase in the word count because of the additional original interview quotes requested by Reviewer 1. We hope the editor agrees that these measures have led to a clearer and more readable manuscript.

For the authors,

Maria Neufeld
