# Supplementary material for: Capturing Russian drinking patterns with the Alcohol Use Disorders Identification Test: An exploratory interview study in primary healthcare and narcology centers in Moscow
Source: PLoS One. 2022 Nov 10;17(11):e0274166. doi: 10.1371/journal.pone.0274166 (PMC9648709; doi:10.1371/journal.pone.0274166)
Supplement: Attachment — Submitted filename: Answers to the reviewers.docx [file pone.0274166.s007.docx]

**PONE-D-21-18136**

**Capturing Russian drinking patterns with the Alcohol Use Disorders Identification Test: an exploratory interview study in primary healthcare and narcology centers in Moscow**

**PLOS ONE**

Additional Editor Comments (if provided):

As I have earlier revised this manuscript I recognize that more citations from interviews are added that makes it easier to follow. The first purpose of the study was to highlight problems that exist with the understanding of the issues in Audit. It is quite clear from the study that the major problems with understanding apply to the concepts of standard drink and also drinking occasion. Thus, the first purpose is achieved. The authors also propose solutions to adapt the issues to the Russian alcohol culture, which was the second purpose.
Whether the third and fourth objectives are achieved is a little more unclear as the author does not discuss different practical solutions for screening and integrating different experiences of Audit.
The tables in the manuscript are not tables but information sheets. Please change the that

I suggest accept after considering this.

**Author response:**

*Dear editor,*

*Thank you very much for the careful consideration of the manuscript.*

*We have included an additional paragraph that discusses the findings of the study in relation to the third and fourth stated objective in greater detail, i.e. the conversion table as a practical solution and its integration with a visual flashcard depicting different beverages as standard drinks (pp. 34-35).We hope that the important implications that this exploratory study has had on the subsequent validation study of the AUDIT in the Russian Federation are now clearer.*

*We have now also revised the paragraph with the sample description (p.14), following earlier reviewer feedback, and have provided the sample size characteristics in-text, thereby removing Table 1 from the manuscript. However, in the current submission guidelines of the journal I could not find any guidance on how information sheets should be handled in the manuscript, following your suggestion to change the tables into information sheet. Please kindly advise on how this should be handled, and I will make the necessary changes within the next 24 hours.*

*Thank you for your considerate feedback*

*With best wishes*

*Maria Neufeld (for the authors)*
