# Supplementary material for: Capturing Russian drinking patterns with the Alcohol Use Disorders Identification Test: An exploratory interview study in primary healthcare and narcology centers in Moscow
Source: PLoS One. 2022 Nov 10;17(11):e0274166. doi: 10.1371/journal.pone.0274166 (PMC9648709; doi:10.1371/journal.pone.0274166)
Supplement: Attachment — Submitted filename: Answers to the reviewers.docx [file pone.0274166.s008.docx]

**PONE-D-21-18136**

**Capturing Russian drinking patterns with the Alcohol Use Disorders Identification Test: an exploratory interview study in primary healthcare and narcology centers in Moscow**

**PLOS ONE**

Additional Editor Comments (if provided):

Dear authors I think it is not correct to use the word table in a qualitative study. I think it is better to delete the words Table 1, Table 2, Table 3 and Table 4. Only the explaining text should remain. After those changes i suggest an acceptance of the manuscript

**Author response:**

*Dear editor,*

*Thank you very much for your considerate feedback. We have now made the suggested changes.*

*With best wishes*

*Maria Neufeld (for the authors)*
